# PW03-035 – Autoinflammatory diseases diagnostic chart/tool

**DOI:** 10.1186/1546-0096-11-S1-A261

**Published:** 2013-11-08

**Authors:** KL Durrant, I Muscari, JI Aróstegui

**Affiliations:** 1 Nursing Adjunct Faculty, Samuel Merritt University, Oakland, USA; 2 Medical & Surgical Sciences and Neuroscience, Institute of Rheumatology, Sienna, Italy; 3 Immunology Department, Hospital Clínic-IDIBAPS, Barcelona, Spain

## Introduction

There is great interest in having a reference chart to increase awareness and improve accurate diagnosis for systemic autoinflammatory diseases (SAID), which arealso classical primary immunodeficiency diseases. We have created this wall chart that includes all currently known SAIDs, and arranged the information so that a clinician could compare various symptoms, abnormal labs, genetics, inheritance pattern, affected ethnicity and other information between all of them. In addition, photographs of the main clinical features of many of these diseases have been included to help medical professionals to better understand these diseases, and consider them in the evaluation of candidate patients. As the understanding of autoinflammatory diseases is continuously changing, this chart will probably need to be updated over time. However, the basic information for these diseases will remain helpful for medical professionals.

## Case report

In 2008, we developed a chart that compared the four main inherited SAID (cryopyrin-associated periodic syndromes [CAPS], TNF receptor-associated periodic syndrome, Familial Mediterranean Fever, and Hyper-IgD periodic fever syndrome). This chart was shared online, in print, and inside our CAPS guidebook available in print or online at http://nomidalliance.org. This chart has helped increase awareness about these diseases. We also received personal feedback from a large number of newly diagnosed patients and families, mostly with CAPS, about the usefulness of this comparative chart in helping their doctors to evaluate their symptoms, make a diagnosis and order genetic testing that later confirmed their disease. Previously undiagnosed families with CAPS in the United States, Australia and elsewhere, many with 6-18 affected family members spanning 3 generations that directly benefitted from downloading the chart from the website. They printed it out, and shared it with their doctor that led to their diagnosis, and prescribed treatment for CAPS. The chart also helped a number of other individuals, including many doctors that were seeking more about these diseases.

## Discussion

We have developed this new, expanded chart including the main features of all currently known SAID. It will be distributed to doctors worldwide to help increase awareness, care and treatment for these diseases. The chart will be translated into various languages, for use online and in print, and will be printed in smaller batches. We can continually adapt it in the online version to keep it as current and accurate as possible. Our hope is that this tool helps to doctors to achieve an early diagnosis in these patients to gain access to treatment, especially at a young age, so that they can have the best chances for a healthy life with less disease complications.

## Disclosure of interest

None declared
